# *Midkine*-Deficiency Delays Chondrogenesis during the Early Phase of Fracture Healing in Mice

**DOI:** 10.1371/journal.pone.0116282

**Published:** 2014-12-31

**Authors:** Melanie Haffner-Luntzer, Aline Heilmann, Anna Elise Rapp, Simon Beie, Thorsten Schinke, Michael Amling, Anita Ignatius, Astrid Liedert

**Affiliations:** 1 Institute of Orthopaedic Research and Biomechanics, University Medical Center Ulm, Ulm, Germany; 2 Department of Osteology and Biomechanics, University Medical Center Hamburg-Eppendorf, Hamburg, Germany; University of Rochester, United States of America

## Abstract

The growth and differentiation factor midkine (Mdk) plays an important role in bone development and remodeling. *Mdk*-deficient mice display a high bone mass phenotype when aged 12 and 18 months. Furthermore, Mdk has been identified as a negative regulator of mechanically induced bone formation and it induces pro-chondrogenic, pro-angiogenic and pro-inflammatory effects. Together with the finding that Mdk is expressed in chondrocytes during fracture healing, we hypothesized that Mdk could play a complex role in endochondral ossification during the bone healing process. Femoral osteotomies stabilized using an external fixator were created in wildtype and *Mdk*-deficient mice. Fracture healing was evaluated 4, 10, 21 and 28 days after surgery using 3-point-bending, micro-computed tomography, histology and immunohistology. We demonstrated that *Mdk*-deficient mice displayed delayed chondrogenesis during the early phase of fracture healing as well as significantly decreased flexural rigidity and moment of inertia of the fracture callus 21 days after fracture. *Mdk*-deficiency diminished beta-catenin expression in chondrocytes and delayed presence of macrophages during early fracture healing. We also investigated the impact of *Mdk* knockdown using siRNA on ATDC5 chondroprogenitor cells *in vitro*. Knockdown of *Mdk* expression resulted in a decrease of beta-catenin and chondrogenic differentiation-related matrix proteins, suggesting that delayed chondrogenesis during fracture healing in *Mdk*-deficient mice may be due to a cell-autonomous mechanism involving reduced beta-catenin signaling. Our results demonstrated that Mdk plays a crucial role in the early inflammation phase and during the development of cartilaginous callus in the fracture healing process.

## Introduction

The growth and differentiation factor midkine (Mdk) has been demonstrated to play an important role in bone development and remodeling [1]–[4]. This 13 kDa heparin-binding molecule, which forms a unique growth factor family together with pleiotrophin, was originally identified to play a pivotal role in neuronal embryonic development [5]. It was subsequently shown that Mdk is expressed during embryonic limb development in the ephiphyseal growth plate [1]. Interestingly, Mdk is rarely expressed in the adult organism, with *Mdk* transcripts found solely in the kidney and long bones [5]. The phenotype of *Mdk*-deficient mice indicated an important role of Mdk in bone development. Our group demonstrated that aged *Mdk*-deficient mice displayed a high bone mass phenotype with increased trabecular bone formation and that an absence of Mdk prevented ovariectomy-induced bone loss [2]. Furthermore, *Mdk*-deficiency had an anabolic effect on cortical bone formation in response to mechanical loading in an *in vivo* ulna-loading model [3]. In addition, we showed *in vitro* that Mdk had a negative effect on Wnt-induced transcription in osteoblastic cells and that it reduced the response of osteoblasts to mechanical loading [3]. The Mdk receptor is supposed to be a complex of the transmembrane receptor-type protein tyrosine phosphatase zeta, LRP6 and the α4β1- and α6β1-integrins [3], [6]. Therefore, Mdk appears to be an inhibitor of osteoblast function, suggesting a negative influence of Mdk on bone remodeling.

Interestingly, Mdk was also expressed during fracture healing in an experimental mouse model of diaphyseal tibial fracture [1]. Mdk expression was detected in mesenchymal stem cells as well as in proliferative and hypertrophic chondrocytes during bone repair. However, little is known regarding the exact role of Mdk in the bone repair process, because there is only this single phenomenological study on Mdk expression during fracture healing.

Bone healing is a complex physiological process, which proceeds via three characteristic stages: the inflammatory, repair and remodeling phases. Mdk may be important for more than one of these phases: Mdk exhibits proinflammatory effects on several immune cells [7], [8] and acts as a proangiogenic factor [9]. Furthermore, a chondrogenic cell line overexpressing Mdk displayed enhanced chondrogenesis [1]. In addition, recombinant Mdk stimulation of osteoblasts reduced Wnt-signaling [3] and increased the expression of several genes, including Ank and Enpp1, which are known to enhance extracellular pyrophosphate, an inhibitor of matrix mineralization [2]. Therefore, Mdk may play a complex role in bone repair regarding its influence on immune cells, vascularization, chondrogenic differentiation and osteoblast function. To evaluate the role of Mdk during fracture healing, we used a standardized femur osteotomy model and analyzed the fracture healing process in *Mdk*-deficient mice. We demonstrated that new bone formation was not influenced by *Mdk*-deficiency, whereas chondrogenesis was delayed. Furthermore, the mechanical properties of the fracture callus were decreased at an early time point of fracture healing in *Mdk*-deficient mice. *In vitro* experiments using Mdk siRNA to knockdown *Mdk* in ATDC5 chondroprogenitor cells showed diminished expression of chondrogenesis-associated genes, which may explain the delay in cartilage formation during fracture repair in Mdk-deficient mice.

## Materials and Methods

### Animal Experiments

All procedures involving animals were in compliance with international regulations for care and use of laboratory animals with the approval of the local ethical committee (No. 1079, Regierungspräsidium Tübingen, Germany). 35 female wildtype littermates and 39 *Mdk*-deficient mice (C57BL/6 genetic background) were used. *Mdk*-deficient mice have been described previously [10]. The mice were maintained in groups of two to four animals per cage (370 cm^2^) on a 14 h light and 10 h dark circadian rhythm with water and food ad libitum.

### Surgical Procedure

Mice received 25 mg/ml tramadol hydrochloride (Tramal, Gruenenthal GmbH, Aachen, Germany) in the drinking water from 1 day pre- to 3 days post-surgery as an analgesia. Surgery was performed under general anesthesia using 2% isoflurane (Forene, Abbott, Wiesbaden, Germany). One dosage of antibiotic clindamycin-2-dihydrogenphospate (45 mg/kg, Ratiopharm, Ulm, Germany) was injected subcutaneously immediately before surgery. The surgical procedure was described previously [11]–[14]. Briefly, the mice received a standardized osteotomy at the diaphysis of the right femur using a 0.4 mm Gigli saw (RISystem, Davos, Switzerland) stabilized using an external fixator (axial stiffness of 3.0 N/mm, RISystem) at the age of 9 months. Osteotomy models are commonly used to model fracture healing, therefore we used the term fracture healing instead of osteotomy healing in this manuscript. The mice were sacrificed 4, 10, 21 or 28 days post-surgery using carbon dioxide. The femurs were carefully explanted for evaluation of fracture healing outcome (n = 6–7 per group and time point).

### Biomechanical Testing

Intact and fractured femurs of mice sacrificed after 21 or 28 days post-surgery were subjected to a nondestructive 3-point-bending test using a materials testing machine as described previously (Z10, Zwick Roell, Ulm, Germany) [11], [12]. After removal of the fixator, the proximal end of the femurs was fixed in an aluminum cylinder using SelfCem adhesive (Heraeus Kulzer, Hanau, Germany). The cylinder itself was fixed in a hinge joint, serving as the proximal support for the bending test. The condyles rested on the distal bending support and the bending load (maximum of 4 N) was applied on top of the cranio-lateral surface of the callus tissue. Flexural rigidity was calculated using the slope of the load-deflection curve. The relative flexural rigidity of the fractured femur was calculated as the ratio between the fractured and contra-lateral femur of the same animal.

### Micro-Computed Tomography (μCT)

After biomechanical testing, the femurs were analyzed using a μCT scanning device (Skyscan 1172, Skyscan, Kontich, Belgium) operating at a resolution of 8 µm and a voltage of 50 kV and 200 mA. Two phantoms with a defined hydroxyapatite density (250 and 750 mg/cm^3^) were scanned within each scan. To determine the total volume (TV), bone volume (BV) and bone volume to total volume fraction (BV/TV), a global threshold of 642 mg hydroxyapatite/cm^3^ according to Morgan et al. [15] was used to distinguish between mineralized and non-mineralized tissue. The following data were obtained without thresholding the tissue: moment of inertia in the anterior-posterior direction (Ix). μCT analysis was performed using Skyscan software (NRecon, DataViewer, CTAn) and in accordance with the American Society for Bone and Mineral Research (ASBMR) guidelines for μCT analysis [16]. Three volumes of interest (VOI) were determined for analysis of the intact or fractured femurs, respectively. The starting point for VOI 1 (trabecular bone) was 200 µm proximal to the metaphyseal growth plate of the intact femur and the endpoint was 160 µm proximal from the starting point. VOI 2 covered the area from 80 µm proximal to 80 µm distal from the middle of the diaphysis of the intact femur. VOI 3 comprised the whole periosteal callus between the two inner pin holes at the fractured femur.

### Histomorphometry of Undecalcified Femurs

After biomechanical testing and μCT-analysis, the femurs explanted 21 and 28 days post surgery were subjected to undecalcified histology by fixation in 4% formalin for a minimum of 48 h, dehydration with ethanol and embedding in methylmethacrylate. Cross-sections of 7 µm thickness were prepared and stained using Giemsa for tissue quantification. The newly formed callus was examined using light microscopy (DMI6000 B, Leica, Heerbrugg, Switzerland) at a 100-fold magnification. The amounts of bone, cartilage, and fibrous tissue were determined using image analysis software (Leica MMAF 1.4.0 Imaging System, Leica, Herbrugg, Switzerland) according to the ASBMR guidelines for bone histomorphometry [17]. To identify osteoclasts, sections were stained for tartrate-resistant acid phosphatase (TRAP) at day 21. Only TRAP-positive cells residing on the bone surface and with more than two nuclei were counted as osteoclasts. For osteoblast counting, sections were stained using Toluidine-blue and the number of osteoblasts was counted under 400-fold magnification using the image analysis software Osteomeasure (OsteoMetrics, Decatur, USA). To determine osteoclast and osteoblast number, a 1.5 mm×0.35 mm rectangular region of interest was evaluated in the middle of the periosteal fracture callus.

### Histomorphometry of Decalcified Femurs and Immunohistochemistry

Fractured femurs of mice sacrificed at 4, 10 or 21 days post-surgery were collected for decalcified histology. The bones were fixed in 4% formalin for 48 h, decalcified using 20% ethylenediaminetetraacetic acid (pH 7.2–7.4) and embedded in paraffin after dehydration. Sections of 7 µm thickness were prepared and stained using Safranin O for histomorphometric analysis. Immunohistochemical staining of Mdk, beta-catenin, dentin matrix protein 1 (Dmp1), ectonucleotide pyrophosphate/phosphodiesterase 1 (Enpp1), platelet endothelial cell adhesion molecule (PECAM) and F4/80 (macrophages) was performed using the following antibodies and dilutions: polyclonal goat anti-mouse Mdk antibody 1∶100 (sc-1398, Santa Cruz Biotechnology, Dallas, USA), polyclonal rabbit anti-mouse β-catenin antibody 1∶150 (AB19022, EMD Millipore Corporation, Merck, Darmstadt, Germany), sheep anti-mouse Dmp1 1∶400 (AF4386, R&D Systems, Minneapolis, USA), goat anti-mouse Enpp1 1∶500 (SAB2500355, Sigma Aldrich, Taufkirchen, Germany), goat anti-mouse PECAM-1 1∶10 (sc-1506, Santa Cruz Biotechnology), rat anti-mouse F4/80 1∶500 (ab6640, AbD Serotec, Kidlington, UK), HRP-conjugated Streptavidin (Zytomed Systems, Berlin, Germany), donkey anti-goat IgG F(ab′)2 biotin-conjugated 1∶100 (sc-3854, Santa Cruz Biotechnology) and goat anti-rat IgG 1∶100 (Invitrogen, ThermoFisher Scientific, Waltham, USA). Respective non-targeting IgGs were used as isotype controls. 3-Amino-9-ethylcarbazol (Zytomed Systems) was used as the chromogen and the sections were counterstained using hematoxylin (Waldeck, Münster, Germany). Slices were analyzed under 200-fold magnification using light microscopy (Leica DMI6000 B, Leica).

### Cell Lines and Culture Conditions

ATDC5, a murine chondroprogenitor cell line, was provided by Sigma-Aldrich (Taufkirchen, Germany). The cells were cultured in a 1∶1 mixture of DMEM and Ham's F12 medium (Gibco, ThermoFisher Scientific, Waltham, USA) containing 5% fetal calf serum (PAA Laboratories, Cölbe, Germany), 1% penicillin/streptomycin (Gibco, ThermoFisher Scientific), 1% L-glutamine (Biochrom, Merck, Darmstadt, Germany), 10 µg/ml human transferrin and 3×10^−8^ M sodium selenite (both Sigma-Aldrich, Taufkirchen, Germany) as described previously [18]. Cells were seeded in 24-well plates at a density of 10.000 cells/cm^2^. To induce chondrogenic differentiation, normal culture medium was supplemented with 10 µg/ml human insulin (Sigma-Aldrich) and 5 ng/ml human TGFbeta1 (R&D Systems, Minneapolis, USA). The cells were maintained at 37°C in a humidified atmosphere under 5% CO_2_. The medium was replaced every 3 days. All experiments were performed in duplicates, three times.

### Real Time – RT-PCR

Total RNA of samples was isolated using the Micro RNeasy Kit (Qiagen, Hilden, Germany) according to manufacturer's instructions and 1 µg of isolated RNA was transcribed into cDNA using the Omniscript Reverse Transcriptase Kit (Qiagen). Quantitative PCR was performed using the Brilliant Sybr Green QPCR Master Mix Kit (Stratagene, Amsterdam, Netherlands) according to the manufacturer's protocol in a total volume of 25 µl using the following cycling conditions: 50°C for 2 min, 95°C for 2 min, 40 cycles each consisting of 95°C for 15 s and 60°C for 1 min. Then melting curve acquisition was performed (95°C for 15 s, 60°C for 1 min, 95°C for 15 s). *B2m* was used as housekeeping gene (F: 5′- CACTGAATTCACCCCCACTGA-3′, R: 5′-TCTCGATCCGTACAGACGGT-3′). Chondrogenic differentiation of ATDC5 cells was analyzed using specific primers for *aggrecan (Acan)* (F: 5′-AACTTCTTTGCCACCGGAGA-3′; R: 5′-GGTGCCCTTTTTACACGTGAA-3′) and *collagen2a1 (Col2a1)* (F: 5′-GGTGCCCTTTTTACACGTGAA-3′; R: 5′-TGGGTATCATCAGGTCAGGT-3′). Primer for beta-catenin-responsive transcription factor L*ef1* were F: 5′-TCATCACCTACAGCGACGAG-3′ and R: 5′-TGACATCTGACGGGATGTGT-3′ and for A*xin2* F: 5′- ATAAGCAGCCGTTCGCGATG-3′ and R: 5′-GCAATCGGCTTGGTCTCTCT-3′. Relative gene expression was calculated using the delta-delta CT method with PCR-efficiency correction using LinRegPCR software as described previously [19].

### RNA Interference of Mdk

RNA interference was performed using siRNA for *Mdk* (AM16708) or nontargeting negative control siRNA (AM4611; both Invitrogen, ThermoFisher Scientific, Waltham, USA). Cells were transfected using Opti-MEM (Gibco, ThermoFisher Scientific) supplemented with 10 µM Lipofectamine RNAiMAX (Invitrogen, ThermoFisher Scientific) and 2,5 pmol Mdk or negative control siRNA for 24 h. The success of *Mdk* knockdown was assessed using real time – RT-PCR and western blotting. Primers for *Mdk* were F: 5′-AGACCATCCGCGTGACTAAG-3′ and R: 5′-GGCTTTGGTCTTTGACTTGG-3′.

### Western Blot Analysis

We resolved 10 µg of cellular lysate protein using SDS-PAGE and transferred it to a nitrocellulose membrane (BioRad, Hercules, USA). The membranes were incubated with anti-GAPDH, Mdk (both Santa Cruz Biotechnology, Dallas, USA), β-catenin (EMD Millipore Corporation, Merck, Darmstadt, Germany) or collagen type 2 (Rockland, Pennsylvania, USA) antibodies overnight at 4°C, respectively. The membranes were incubated using horseradish peroxidase-conjugated secondary antibodies and developed in SuperSignal West Pico Chemiluminescent Substrate (Perbio Science, ThermoFisher Scientific, Waltham, USA). The protein bands were visualized using the Fusion Molecular Imaging System (Vilber Lourmat, Eberhardzell, Germany).

### Statistical Analysis

Sample size was calculated based on a previous fracture healing study for the main outcome parameter flexural rigidity in the fractured femur (power: 80%, alpha = 0.05) [14].

The data were analyzed for significance by a non-parametric Mann-Whitney-U test using SPSS software (SPSS Inc., Chicago, USA). The level indicating significance was assumed at p<0.05. The results were displayed as box plots with median, first and third quartiles, and maximum and minimum values. Outliers (value is less than the first quartile minus 1.5 times the interquartile range or greater than the third quartile plus 1.5 times the interquartile range) were marked as circles.

## Results

### Increased Trabecular Number and Decreased Cortical Thickness in Mdk-Deficient Mice Aged 9 Months

**Figure 1:**
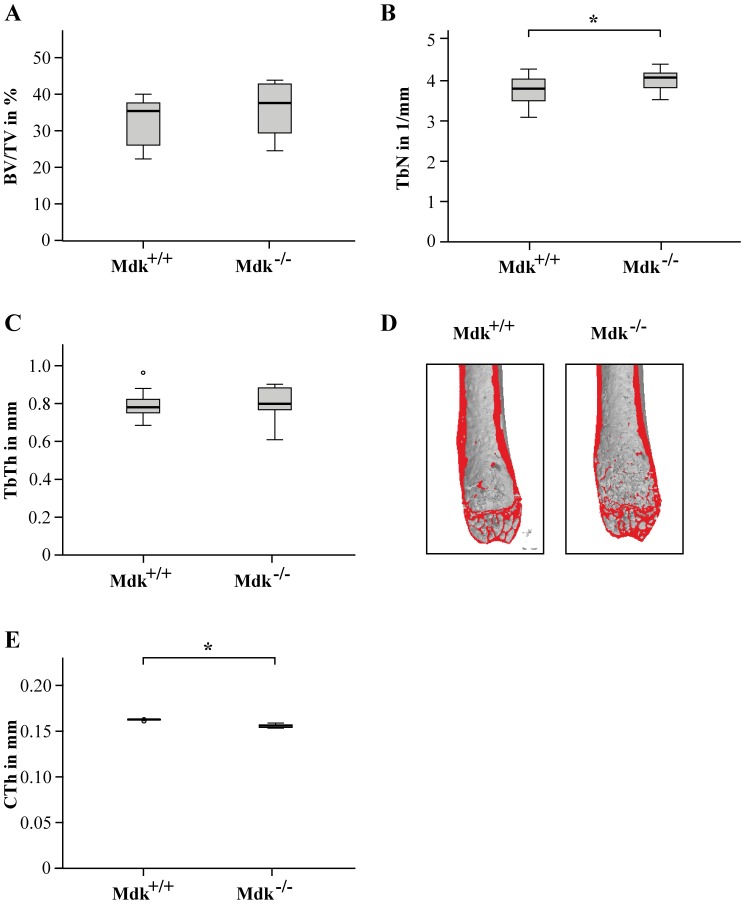
*Mdk*-deficient mice aged 9 months displayed increased trabecular number and decreased cortical thickness in the femur. A) Trabecular bone volume to tissue volume ratio assessed by μCT analysis of volume of interest (VOI) 1 in the intact femur. B) Trabecular number assessed by μCT analysis of the VOI 1 in the intact femur. C) Trabecular thickness. D) Representative μCT images of the intact femurs. E) Cortical thickness assessed by μCT analysis of VOI 2 in the intact femur. *Significantly different from wildtype (p<0.05). (n = 6–7 per group).

Because it has been shown by our group that aged *Mdk*-deficient mice displayed increased trabecular bone formation and decreased cortical thickness at 12 month of age [2], we analyzed the trabecular and cortical bone phenotypes of wildtype and *Mdk*-deficient mice aged 9 months (Fig. 1). Although we found no significant differences in trabecular thickness and trabecular BV/TV, the trabecular number was significantly increased whereas the cortical thickness was significantly decreased. These results demonstrate that 9-month-old *Mdk*-deficient mice displayed a similar bone phenotype in the femur, but less distinct than the phenotype of 12-month-old mice.

### Mdk-Deficient Mice Exhibited Decreased Bone Rigidity during Fracture Healing

**Figure 2:**
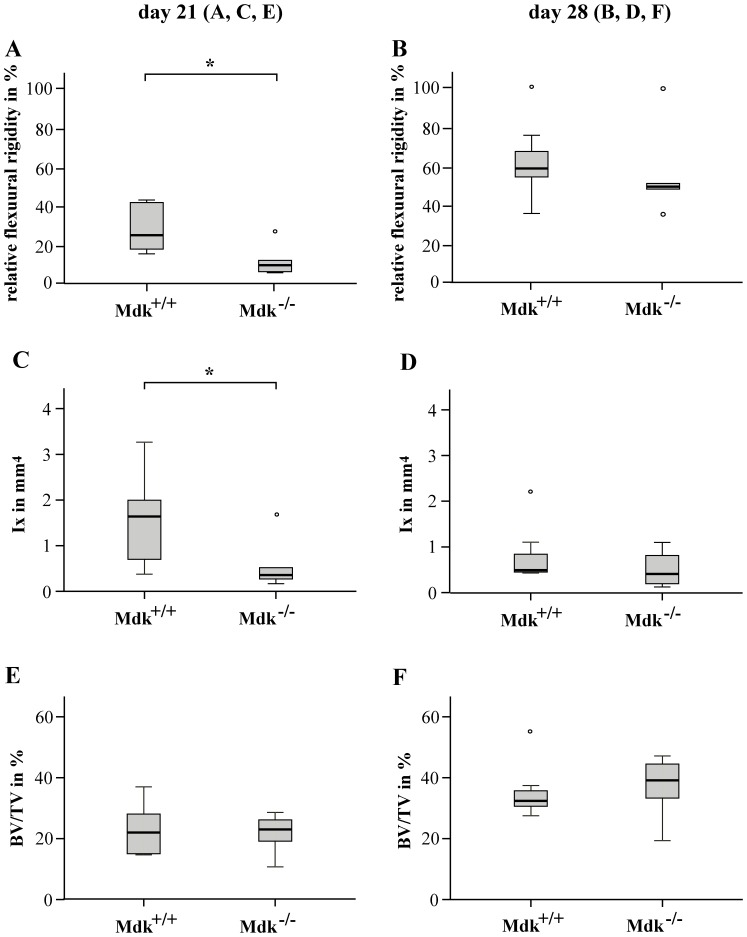
Flexural rigidity and moment of inertia was significantly reduced in *Mdk*-deficient mice after 21 days of the healing period. Biomechanical and μCT analyses of fractured femur after 21 and 28 days of healing. The volume of interest was determined as the whole periosteal callus between the two inner pin holes. A) Relative flexural rigidity of fractured femur in comparison to intact femur at day 21 and B) at day 28. C) Moment of inertia of the periosteal fracture callus in bending axis x at day 21 and D) at day 28. E) Bone volume to total volume fraction of the periosteal fracture callus at day 21 and F) at day 28. *Significantly different from wildtype (p<0.05). (n = 6–7 per group).

To evaluate the fracture healing after 21 and 28 days, the femurs of wildtype and *Mdk*-deficient mice were analyzed for their mechanical competence and bone content using 3-point bending and μCT analysis, respectively (Fig. 2). Animals lacking *Mdk* displayed a significantly decreased relative flexural rigidity of the fractured femur 21 days after surgery (Fig. 2A). The BV/TV in the periosteal callus at this time point did not differ significantly from wildtype littermates (Fig. 2E). Because flexural rigidity does not only depend on the quality of the callus tissue but also on callus geometry, the moment of inertia was determined using μCT analysis. In *Mdk*-deficient mice, the moment of inertia in the bending axis (Ix) was significantly reduced at day 21 (Fig. 2C). After 28 days of healing, the mean flexural rigidity of the fractured femurs of both genotypes attained approximately 55–60% of the intact femurs. There was no significant difference between both genotype groups regarding flexural rigidity, moment of inertia or BV/TV of the fractured femur (Fig. 2B, D, F). Therefore, fracture healing was altered in the absence of *Mdk* at an earlier time point (d21), but not at the late stage of fracture healing (d28).

### Absence of Mdk Altered Cartilage Formation

**Figure 3:**
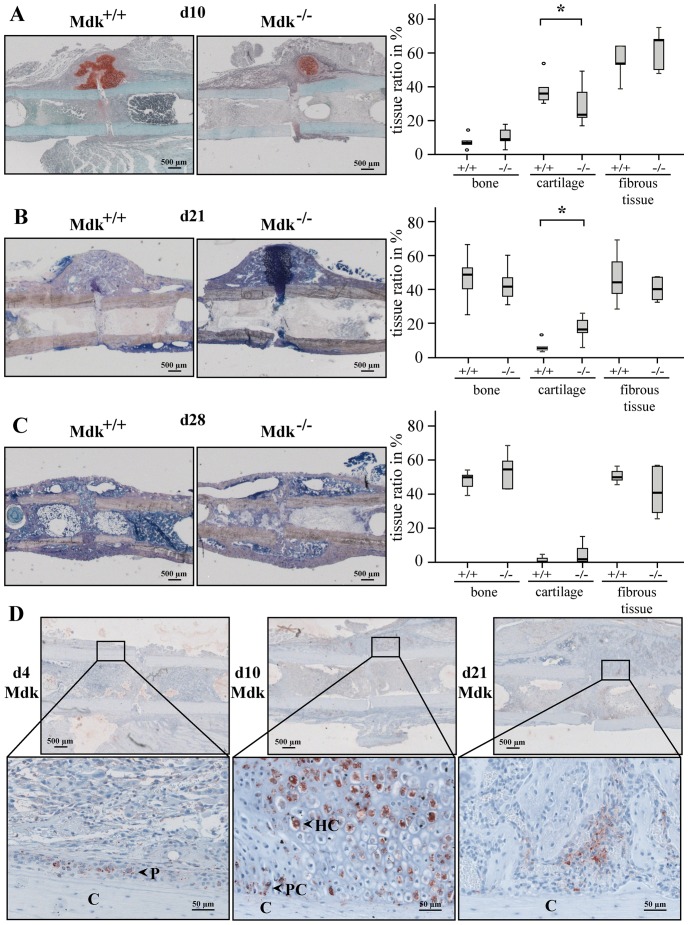
*Mdk*-deficient mice showed significantly less callus cartilage than wildtype mice 10 days after fracture and significantly more cartilage 21 days after fracture. Histological sections of fractured femurs were analyzed histomorphometrically using A) Safranin O staining at day 10 or B) Giemsa staining at day 21 and C) at day 28. Scale bar: 500 µm. D) Immunohistochemical Mdk staining of the periosteal callus of wildtype mice at day 4, 10 and 21, showing positively stained periosteal cells at day 4, proliferating and hypertrophic chondrocytes at day 10 and Mdk staining at day 21 in areas of new bone formation. C = cortex; P = periost; PC = proliferating chondrocyte; HC = hyperthropic chondrocyte. Representative images are shown. Scale bar: 500 µm at the upper panel and 50 µm at the lower panel. *Significantly different from wildtype (p<0.05). (n = 6–7 per group).

For further evaluation of the fracture healing progress, we analyzed the exact composition of the fracture callus using histomorphometry (Fig. 3A–C). We observed that *Mdk*-deficient mice displayed significantly decreased cartilage in the newly formed callus after 10 days whereas the cartilage content was significantly increased after 21 days (Fig. 3A, B). The amount of bone or fibrous tissue was not significantly altered at day 10 or 21. At day 28, there was no significant difference between the two genotype groups in the amount of bone, cartilage or fibrous tissue (Fig. 3C).

### Mdk Was Expressed during the Early and Intermediate Phase of Fracture Repair

We analyzed the spatio-temporal pattern of Mdk expression during fracture repair. Immunohistochemical staining for Mdk was performed at 4, 10 and 21 days (Fig. 3D). Little Mdk was expressed 4 days after fracture; there were few positively stained cells in the periosteal region. The peak Mdk expression was reached at day 10 with strong expression in the cytoplasm of proliferating and hypertrophic chondrocytes. On day 21, most chondrocytes were replaced by newly formed bone. Mdk expression was only observed in scattered chondrocytes between the newly formed bone trabeculae as well as in some areas of neovascularization and new bone formation at the periosteal callus.

### Expression Level of Beta-Catenin Was Decreased in Mdk-Deficient Animals

**Figure 4:**
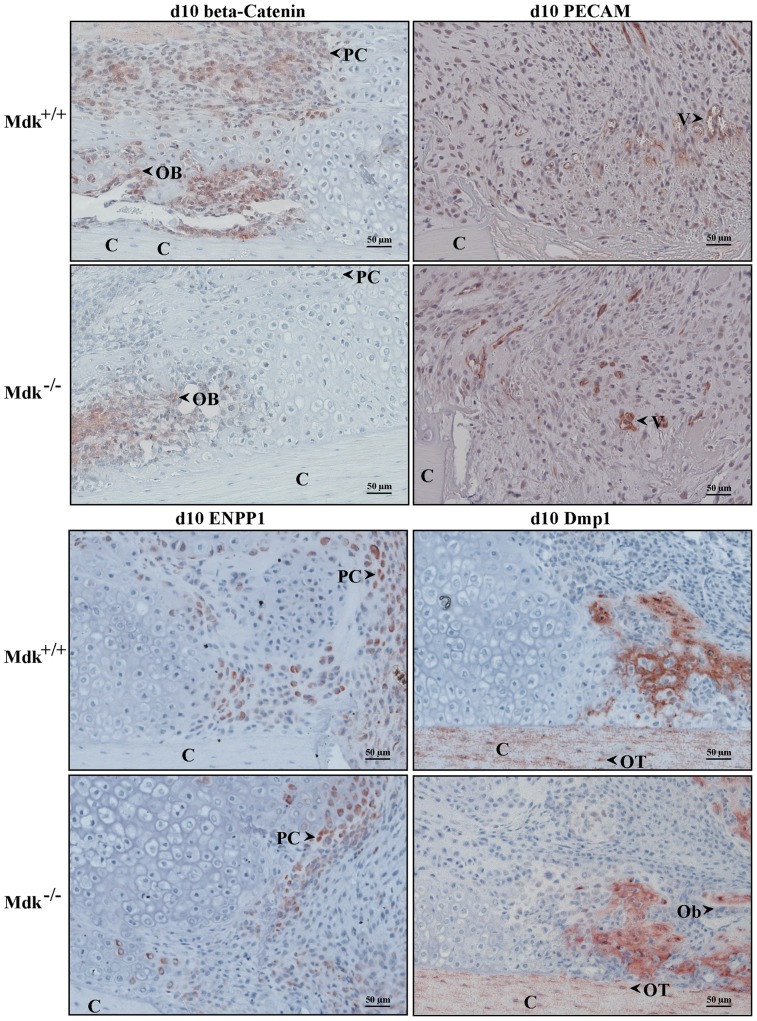
Immunohistochemical staining showing reduced beta-catenin levels in chondrocytes of *Mdk*-deficient mice. Sections of fractured femurs from four mice of each time point and genotype group were stained for each antigen and counterstained using hematoxylin. Representative images are shown; C = cortex; PC = proliferating chondrocyte; Ob = osteoblast; V = vessel; OT = osteocyte; scale bar 50 µm; 200-fold magnification. Beta-catenin staining of the periosteal callus at day 10. PECAM staining of the periosteal fracture callus bridging the osteotomy gap showing the endothelial cells of the newly formed vessels in an area of proliferating chondrocytes at day 10. Enpp1 staining of the osteotomy gap at day 10 showing positively stained proliferating chondrocytes. Dmp1 staining of the periosteal callus at day 10 showing positively stained cortex, osteocytes and areas of new bone formation. Osteoblasts were Dmp1 negative. (n = 4 per group).

Because Mdk is considered to be an inhibitor of Wnt/beta-catenin signaling in osteoblasts, we investigated the presence of beta-catenin during fracture healing in wildtype and *Mdk*-deficient mice. While beta-catenin expression was found in osteoblasts and proliferating chondrocytes, it was absent in hypertrophic chondrocytes. We found clear differences between the two genotypes. Beta-catenin expression was less intense in proliferating chondrocytes of *Mdk*-deficient mice, suggesting an influence of *Mdk*-deficiency on Wnt-signaling in these cells (Fig. 4). In contrast, beta-catenin expression was similar in the osteoblasts at the areas of intramembranous bone healing, indicating a different influence of Mdk on this protein in both cell types.

Mdk is known to stimulate angiogenesis [9]. To test whether *Mdk*-deficiency influences neovascularization during fracture healing, we analyzed newly formed blood vessels in the fracture callus. Endothelial cells were stained immunohistochemically using an anti-PECAM antibody. In both wildtype and *Mdk*-deficient mice new blood vessels were detected 10 days after fracture in the periphery of the callus and in the area of proliferating chondrocytes near the osteotomy (Fig. 4). There were no distinct differences in PECAM staining between the genotype groups.

It was demonstrated in *in vitro* osteoblast cultures that Mdk regulates the expression of several genes, which are related to matrix mineralization [2]: Dmp1, an acidic phosphorylated extracellular matrix protein located in mineralized tissue [20] and Enpp1, a regulator of tissue mineralization by controlling the extracellular levels of inorganic pyrophosphate [21]. To determine whether *Mdk*-deficiency influences the expression of these proteins during fracture healing, immunohistochemical staining for these proteins were performed at days 10 and 21 (Fig. 4). No distinct differences in the expression levels of both proteins were found between wildtype and *Mdk*-deficient mice at both time points. At day 10, Dmp1 was highly expressed in cortical bone and in newly formed bone trabeculae around the osteocytes. The location of Dmp1 expression was similar at day 21, however, the expression level was generally lower (data not shown). Enpp1 was highly expressed in proliferating chrondrocytes in and near the osteotomy gap at day 10, whereas its expression was lower in more mature chondrocytes and absent in hypertrophic chondrocytes in the periosteal callus. At day 21, most chondrocytes were replaced by newly formed bone, therefore, Enpp1 expression was very limited (data not shown). Enpp1 expression was slightly lower in *Mdk*-deficient mice at day 10 because of the reduced number of chondrocytes in these animals.

### Presence of macrophages was delayed in Mdk-deficient mice

**Figure 5:**
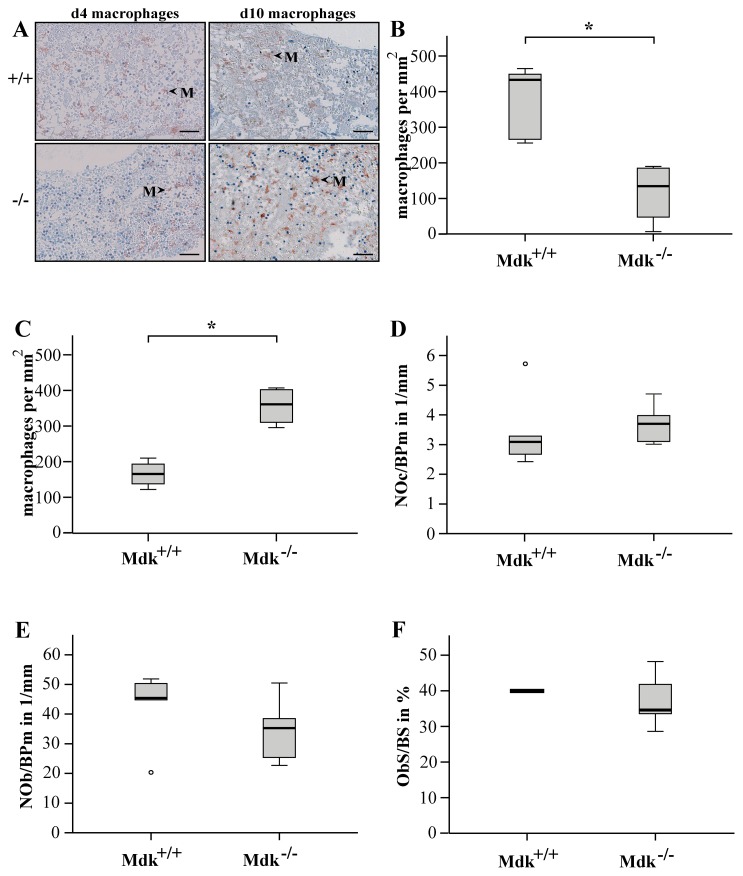
Presence of macrophages was delayed in *Mdk*-deficient mice. A) Immunohistochemical staining for macrophages at days 4 and 10. Representative images showing recruited macrophages in the marrow cavities proximal to the osteotomy gap. M = macrophage; scale bar 50 µm; 200-fold magnification. The number of macrophages was counted in the marrow cavities close to the osteotomy gap B) at day 4 and C) at day 10. D) TRAP-stained sections from fractured femurs were analyzed for the number of osteoclasts per bone perimeter. E) Toluidine-blue-stained sections were analyzed for the number of osteoblasts per bone perimeter and F) Osteoblast surface per bone surface. (n = 5–6 per group).

Mdk stimulates macrophage migration [22], thus we analyzed the numbers of macrophages during the early stage of fracture healing in wildtype and *Mdk*-deficient mice. After 4 and 10 days, macrophages were found mainly in the marrow cavities near the osteotomy gap. *Mdk*-deficient mice showed significantly less macrophages compared to wildtype animals at day 4 but more macrophages at day 10 (Fig. 5A–C).

It was previously shown that *Mdk*-deficient mice displayed increased numbers of osteoblasts and decreased numbers of osteoclasts in the trabecular bone of the vertebral bodies and increased numbers of osteoclasts in the cortical bone of the tibia [2]. Therefore, we analyzed the amount of these cell types in the periosteal callus at day 21. We found no significant differences with regard to osteoclast or osteoblast number, or of the osteoblast surface to bone surface ratio between the two genotypes on day 21 (Fig. 5D–F). Therefore, *Mdk*-deficiency did not significantly influence the differentiation of osteoclasts and osteoblasts during fracture healing.

### Mdk-Knockdown Delayed Chondrogenic Differentiation *In Vitro*

**Figure 6:**
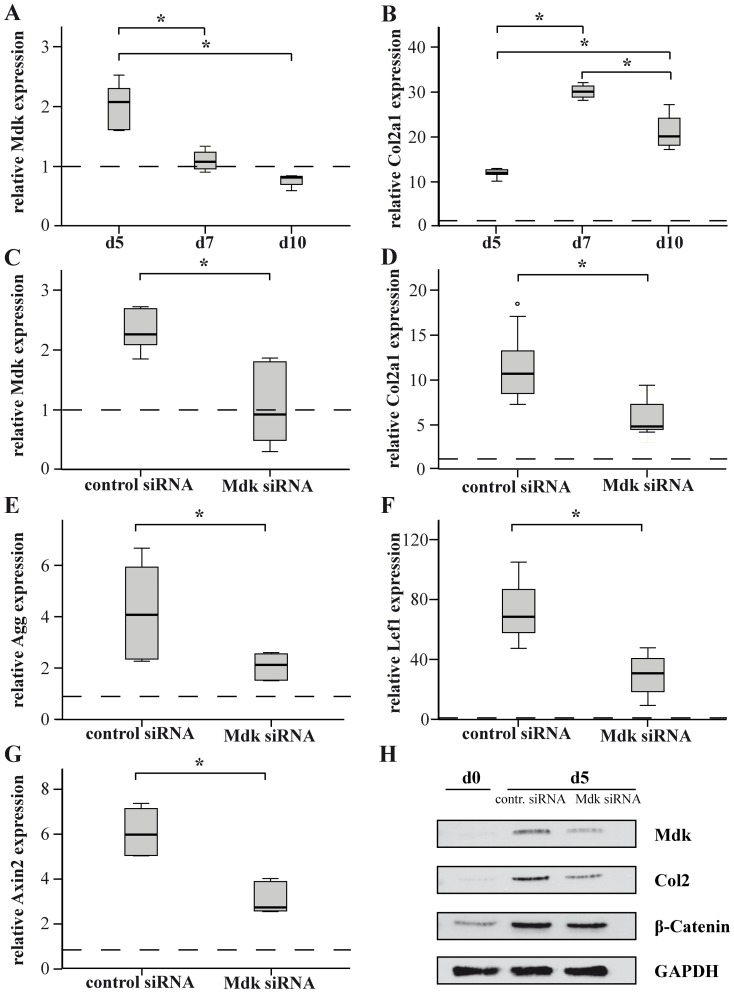
*Mdk* is expressed during ATDC5 cell differentiation and *Mdk* knockdown significantly delayed early chondrogenic differentiation via suppression of Wnt-target genes. ATDC5 cells were differentiated and gene expression was evaluated using real-time RT-PCR. *B2M* was used as the housekeeping gene and gene expression values were normalized to the pre-differentiation values (dotted line). ATDC5 cells were incubated in differentiation medium for 5, 7 and 10 days and A) *Mdk* and B) *collagen2a1* gene expression was evaluated using real time RT-PCR. C) ATDC5 cells were incubated with control siRNA or Mdk siRNA for 24 h and subsequently differentiated for 5 days. *Mdk* knockdown was verified by analyzing *Mdk* gene expression. Differentiation was analyzed by evaluation of D) *aggrecan* or E) *collagen2a1* gene expression. Beta-catenin signaling was analyzed by evaluation of F) *lef1* and G) *axin2* gene expression. H) Western blot analysis of Mdk, collagen type 2 and beta-catenin protein expression at days 0 and 5 of differentiation. GAPDH was used as control. *Significantly different from the control values (p<0.05). (n = 6 per group).

Because delayed chondrogenesis appeared to be the predominant effect of *Mdk*-deficiency on fracture healing, we next performed *in vitro* experiments using ATDC5 chondroprogenitor cells to determine whether *Mdk*-deficiency influences chondrogenic differentiation in a cell-autonomous manner. For this purpose, ATDC5 cells were incubated in chondrogenic differentiation medium for 10 days. *Mdk* expression was upregulated on day 5 during chondrogenic differentiation (Fig. 6A, B). To analyze the effects of *Mdk* knockdown on chondrocyte differentiation, the cells were transfected using nontargeting control siRNA or *Mdk* siRNA and differentiated for 5 days (Fig. 6C–H). *Mdk* knockdown significantly decreased *aggrecan* and *collagen2a1* gene expression (Fig. 6D, E). The expression of the beta-catenin target genes *lef1* and *axin2* [23] was also significantly decreased (Fig. 6F, G). Protein expression analysis demonstrated that *Mdk* knockdown decreased both collagen type 2 and beta-catenin protein expression (Fig. 6E).

## Discussion

In this study we analyzed the influence of *Mdk*-deficiency on fracture healing in mice. We showed that an absence of Mdk led to delayed early fracture healing due to retarded cartilage formation in the periosteal callus. Moreover, we demonstrated *in vitro* using ATDC5 chondroprogenitor cells that *Mdk* knockdown led to decreased chondrogenic differentiation, suggesting that Mdk plays an important role during the development of the cartilaginous callus.

Our main objective in this study was to determine whether fracture healing was influenced by *Mdk*-deficiency. Biomechanical testing demonstrated decreased callus rigidity in *Mdk*-deficient mice at an earlier time point during fracture healing, it was not affected during the late stage. The lower biomechanical stability of the fractured bone resulted from a significantly decreased moment of inertia of the fracture callus, because flexural rigidity does not only depend on the quality of the callus tissue but also on callus geometry. The bone volume ratio was similar in the fracture callus of both genotype groups. We expected that an absence of Mdk would promote osteoblast activity, because Mdk has been suggested to be an inhibitor of Wnt signaling in osteoblasts [3], and it has been shown for Dkk-1 and SOST, two Wnt signaling inhibitors, that osteoblast activity and therefore fracture healing was accelerated by antagonizing these two proteins [24], [25]. However, our data showed no differences in bone formation in the fracture callus at all analyzed time points. In contrast, the amount of cartilage was altered in the fracture callus of *Mdk*-deficient animals. We demonstrated that *Mdk*-deficient mice exhibited less cartilage at day 10, but more cartilage at day 21 after surgery compared to wildtype animals. Therefore, cartilage formation during endochondral ossification was delayed in *Mdk*-deficient mice, causing delayed early fracture healing. The previous findings that Mdk overexpression in a chondrogenic cell line resulted in enhanced chondrogenesis [1] and that systemic delivery of recombinant Mdk led to an increase in knee cartilage in rats [26] provide further evidence that Mdk is essential for the normal development of cartilaginous callus and that a lack of Mdk has a significant influence on chondrocytes during fracture healing. Because it has been shown that Mdk is expressed in chondrocytes during tibial fracture healing in an ICR mouse model [1], we next analyzed the spatio-temporal expression pattern of Mdk during callus development. We were able to confirm earlier data and demonstrated that Mdk was highly expressed in chondrocytes during early fracture healing. This finding further corroborated our theory that Mdk plays an important role during chondrogenesis in fracture healing.

As Mdk is considered to be an inhibitor of the Wnt signaling pathway in osteoblasts [3], we investigated the influence of *Mdk*-deficiency on beta-catenin expression during fracture healing. We demonstrated that the level of beta-catenin expression in osteoblasts was unaffected by *Mdk*-deficiency, whereas its expression was lower in chondrocytes of *Mdk*-deficient mice, indicating a different influence of Mdk on these cell types. It was previously shown, that inhibition of beta-catenin signaling in chondrocytes induced delayed fracture healing based on impaired chondrogenesis in mice [27]. Thus, the lower expression of beta-catenin in chondrocytes of *Mdk*-deficient animals could be one explanation for the delayed chondrogenesis during endochondral ossification and therefore the delayed early fracture healing. To further investigate the unexpected lack of influence of *Mdk*-deficiency on osteoblast function during fracture healing, we focused on the expression of several genes, which are known to be influenced by Mdk and are associated with osteoblastic differentiation [2]. A previous study had shown that treatment of osteoblasts with recombinant Mdk upregulated the expression of Dmp1, an acidic phosphorylated extracellular matrix protein located in mineralized tissue [20], and Enpp1, a regulator of tissue mineralization [21]. Dmp1 has been demonstrated to play an important role in phosphate homeostasis and *Dmp1* knockout mice displayed disturbed matrix mineralization [28]. *Enpp1* knockout caused ectopic calcification in mice due to decreased levels of pyrophosphate, an inhibitor of matrix mineralization [29]. The data of the present study provide evidence that a lack of Mdk did not significantly influence the expression of these proteins during early and intermediate fracture healing. This could be an explanation for the missing effects of *Mdk*-deficiency on bone formation during fracture healing.

Because Mdk is known to have angiogenic potential [9], we also visualized the newly formed blood vessels in the fracture callus using PECAM immunostaining. We demonstrated that *Mdk*-deficiency did not alter blood vessels formation in the periosteal callus. It was shown previously that hind-limb ischemia induced Mdk expression in endothelial cells and that *Mdk*-deficient mice showed almost no neovascularization in response to this hypoxic condition [9]. However, in that ischemia model, hypoxia was present for 4 days, whereas local hypoxia occurs in fracture healing for <1 day [30]. This could explain the lack of vascularization-related differences between wildtype and *Mdk*-deficient mice in the course of fracture healing. Moreover, we could not detect any Mdk expression in endothelial cells at 4 days after injury.

It was previously shown that *Mdk*-deficient mice exhibited significantly fewer macrophages in skeletal muscle after injury and in renal tissue during tubulointerstitial inflammation associated with diabetic nephropathy [22], [31]. We were able to show that the presence of macrophages was significantly delayed in *Mdk*-deficient mice during the early phase of fracture healing possibly due to delayed recruitment of these cells. Although the role of macrophages in fracture healing had not been clearly investigated, there are indications that they have an important function in bone healing. It was previously shown that the depletion of osteomacs and macrophages significantly repressed new bone formation and mineralization during intramembranous bone healing [32]. Another study demonstrated that delayed macrophage recruitment following fracture resulted in delayed cartilage maturation and decreased callus size [33]. Therefore, the attenuated migration of macrophages to the injury site could be another mechanism of delayed early fracture healing in *Mdk*-deficient mice due to the influence of macrophages on cartilage formation.

Because it has been shown previously that *Mdk*-deficient mice exhibited an increased trabecular number and reduced cortical thickness when aged 12 months accompanied by a greater number of trabecular osteoblasts and cortical osteoclasts [2], we also investigated the number of osteoblasts and osteoclasts in the fracture callus. We did not observe significant differences between wildtype and *Mdk*-deficient mice, just a trend towards increased osteoclast number, suggesting that the *Mdk*-deficiency did not significantly influence the differentiation of osteoblasts during fracture healing. Regarding the intact skeleton of the *Mdk*-deficient mice, we showed that the 9-months-old *Mdk*-deficient mice also exhibited an increased trabecular number and decreased cortical thickness in the intact femur. Therefore, we concluded that the previously published influence of *Mdk*-deficiency on osteoblasts and osteoclasts is responsible for the bone phenotype of the mice in the intact skeleton [2], whereas the negative influence of *Mdk*-deficiency on the development of the cartilaginous callus is predominantly responsible for the delayed early fracture healing demonstrated in this study at day 21. However, interestingly we found no delay in remodeling of the cartilaginous callus to bony tissue at day 28, suggesting that *Mdk*-deficiency did not only delayed chondrogenesis during the early phase of fracture healing but also sped up bone remodeling of the callus cartilage at the later phase of fracture healing possibly due to the previously published high bone turnover phenotype in Mdk-deficient mice. The trend towards an increase in osteoclast number at day 21 corroborates this hypothesis. However, since our mouse model displayed a systemic *Mdk*-deficiency, we could not clearly discriminate between the influence of *Mdk*-deficiency on osteoblast, osteoclasts or chondrocytes during fracture healing. Therefore and because of the demonstrated delay in chondrogenesis, we finally investigated the direct influence of *Mdk*-deficiency on chondrocytes *in vitro*. It has been shown previously that recombinant Mdk had a direct proliferative effect on rat primary articular chondrocytes *in vitro* [26] and that Mdk overexpression resulted in an accelerated differentiation of ATDC5 chondroprogenitor cells [1]. We demonstrated that *Mdk* knockdown significantly decreased the expression of differentiation-related matrix molecules like type II collagen in ATDC5 cells at the early stage of chondrogenic differentiation. Furthermore, the protein expression of beta-catenin was decreased after *Mdk* knockdown. It has been shown previously, that inhibition of Wnt/beta-catenin signaling decreased the expression of collagen type II as well as proliferation and differentiation of chondrocytes *in vivo* [27], [34]. We also demonstrated that the expression of the beta-catenin target genes *lef1* and *axin2* [23] was significantly reduced after *Mdk* knockdown. Axin2 was shown to be involved in chondrogenesis and Axin2-deficient mice displayed depressed collagen type II levels [35]. These findings further corroborated our hypothesis that *Mdk*-deficiency led to delayed chondrogenesis based on reduced beta-catenin signaling in chondrocytes during early fracture healing and that Mdk had a different influence on beta-catenin signaling in chondrocytes as it was reported for osteoblasts [3].

For further evaluation of the exact role of Mdk in physiological fracture healing, the application of a specific Mdk antagonist during fracture healing is proposed, because the bone phenotype was already altered in the *Mdk*-deficient mice aged 9 months in the present study. Therefore, the molecular mechanisms of fracture healing in *Mdk*-deficient mice may differ from those in wildtype mice because of an adaption to the complete absence of Mdk during skeletal development.

In conclusion, the findings of the present study indicate that Mdk plays an important role in the early inflammation phase and endochondral ossification during fracture healing. *Mdk*-deficiency significantly delayed presence of macrophages and chondrogenesis during the early phase of bone repair.
